# Erosion of tropical bird diversity over a century is influenced by abundance, diet and subtle climatic tolerances

**DOI:** 10.1038/s41598-021-89496-7

**Published:** 2021-05-11

**Authors:** Jenna R. Curtis, W. Douglas Robinson, Ghislain Rompré, Randall P. Moore, Bruce McCune

**Affiliations:** 1 Department of Fisheries, Wildlife and Conservation Sciences, Oregon State University, 104 Nash Hall, Corvallis, OR 97331 USA; 2 Department of Botany and Plant Pathology, Oregon State University, 2082 Cordley Hall, Corvallis, OR 97331 USA

**Keywords:** Ecology, Biogeography, Community ecology, Conservation biology, Tropical ecology

## Abstract

Human alteration of landscapes leads to attrition of biodiversity. Recommendations for maximizing retention of species richness typically focus on protection and preservation of large habitat patches. Despite a century of protection from human disturbance, 27% of the 228 bird species initially detected on Barro Colorado Island (BCI), Panama, a large hilltop forest fragment isolated by waters of Gatun Lake, are now absent. Lost species were more likely to be initially uncommon and terrestrial insectivores. Analyses of the regional avifauna, exhaustively inventoried and mapped across 24 subregions, identified strong geographical discontinuities in species distributions associated with a steep transisthmian rainfall gradient. Having lost mostly species preferring humid forests, the BCI species assemblage continues to shift from one originally typical of wetter forests toward one now resembling bird communities in drier forests. Even when habitat remnants are large and protected for 100 years, altered habitat characteristics resulting from isolation produce non-random loss of species linked with their commonness, dietary preferences and subtle climatic sensitivities.

## Introduction

Deforestation, fragmentation, and habitat degradation are among the greatest threats to biodiversity in species-rich lowland Neotropical rainforests^1–3^. Human disturbance disproportionately targets lowland habitats^4^ creating isolated, resource-limited hilltop forest fragments throughout the Neotropics^5^. Species traits also influence extinction risk in isolated fragments. Among birds, species at highest risk are hypothesized to be dietary specialists with small populations^6–9^, large bodies^10,11^ and/or species that experience greater nest predation^12^. In fragments, greater light penetration, increased ambient temperatures, and desiccation by wind can lead to localized climatic change and alteration of microhabitat availability^13–16^. Forest-dwelling birds specialized to low light conditions below dense canopy foliage^17,18^ and having narrow physiological tolerances may be susceptible to disrupted humidity and temperature regimes^19–21^. Yet, even when conditions deteriorate, populations may persist for many decades in large remnant habitats because the time required to fully express expected extinction rates is positively related to size of the habitat patch and initial population size^22–24^. Most attempts to elucidate drivers of species loss in tropical forest fragments have involved studies of smaller patches (< 200 ha) and shorter time spans (< 40 years)^9,13,17,25–31^, so may detect fast-acting mechanisms of extinction but under-value or overlook those that require much longer to appear.

A century-long history of ornithological surveys in humid forests of southern Central America provides an opportunity to identify predictors of species extirpation following habitat isolation. Barro Colorado Island (BCI) has been isolated since 1914 from nearby lowland forests of central Panama following construction of the Panama Canal and filling of Gatun Lake. At 1562 ha, BCI is the largest island in Gatun Lake and the most protected from human disturbance. Protection from human activities is effective enough for BCI to support sizable populations of gallinaceous birds, such as Crested Guan (*Penelope purpurascens*; see Supplementary Table S3 for scientific names), which have been hunted to extirpation elsewhere in the region. Its semi-deciduous lowland forest varies from 100 to > 500 years old, the eastern and youngest half having matured since prior cultivation was eliminated, leading to 99% forest cover today^32,33^. Situated mid-way along a strong north–south precipitation gradient across the isthmus, the average annual precipitation (2600 mm) is two-thirds that of the wettest central Panama forests and double the driest ones^34^. Total annual precipitation on the island has remained relatively consistent^35^ though several extended periods of below-average precipitation occurred in the past century^36^.

Uniquely, the BCI bird community has been inventoried by highly skilled ornithologists for 90 years and sits amidst one of the most thoroughly surveyed regions for birds in all the tropics^3,37^. The long history of bird study in the region provides an unprecedented level of detail on species’ distributions, habitat associations, natural and life histories and abundances. Earlier surveys documented enduring avian species losses from BCI following its isolation^38–40^ which could not be fully explained by habitat loss or classic extinction hypotheses such as habitat succession, nest depredation or mesopredator release^41–43^. Previously described losses of early successional species are easily explained^39,41^, but the loss of forest-dwelling species when tall forest on the island has effectively doubled in extent during the last century is particularly puzzling.

We identified attributes associated with the century-long record of avian extinctions to characterize shifts in community composition and evaluate predictors of fragmentation sensitivity among forest-dwelling bird species. We also developed a novel variable to assess a species’ sensitivity to forest moisture conditions, a previously untested driver of species losses^38^. To further evaluate the degree to which community change is influenced by reduced access to humid forest, we compared BCI species inventories over the last century to regional bird communities whose species composition varies predictably across central Panama’s strong north–south precipitation gradient^3,37^.

Our evaluation of changes in the forest-dwelling avifaunal assemblage on BCI revealed disproportionate losses of species occurring primarily in the most humid forests of central Panama. Loss of moisture-sensitive species suggests subtle climatic change contributes to elevated extinction risk alongside other factors such as abundance, diet, and habitat isolation, even in an otherwise undisturbed forest fragment.

## Methods

We obtained data from our own annual surveys (1994 to 2018) and from published lists of birds observed on BCI by highly skilled ornithologists from 1925 to 1994^39,40,44–48^. Birds were inventoried using a variety of methods, from species lists collected during short visits to abundance estimates derived from comprehensive stationary point and transect counts. All surveys incorporated ad lib observations to some degree. Presence/absence data were used because not all survey periods collected abundance information and the accuracy of such count data is difficult to quantify^48^. We compiled these published observation records into lists of species present in seven non-overlapping time-periods: 1925–1929, 1930–1937, 1938–1951, 1953–1969, 1970–1978, 1990–2000, and 2001–2020. Because bird inventory effort varied across years these “binned” years represent time periods during which particular observers were actively surveying the bird community. We generated an additional list of BCI species we predict to disappear within the next 20 years because we know they currently have low abundances, have been declining since the 1970s and share traits with other species that have already disappeared. Species not reported as present during a given period were assumed absent. Several common species known to be difficult to detect were missing from earlier datasets, likely due to incomplete knowledge of bird species at the time. We evaluated how those missing species affected our statistical results (Supplementary Information, Likely Species).

**Figure 1:**
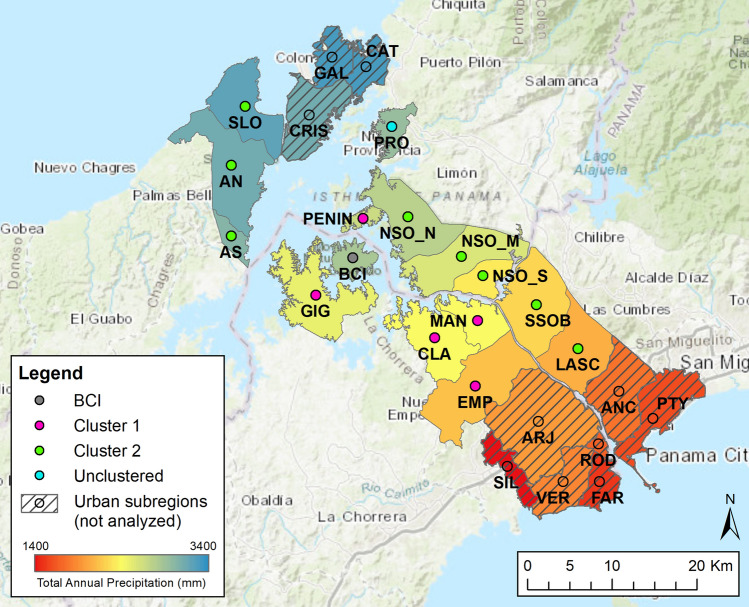
Digitized map of physiographic subregions along the Panama Canal modified from^37^. Colored dots indicate subregion group membership based on hierarchical cluster analysis (Fig. 2). Black lines indicate regions used for the study, with fill colors corresponding to mean total annual precipitation for that subregion. See Supplementary Table S7 for full names corresponding to abbreviated subregion codes.

To provide regional context for BCI, we incorporated bird inventory data from Panama Canal-adjacent “subregions” defined by political administration as well as topographic complexity, geology, and elevation^37^; (Fig. 1). We limited our scope of investigation to only those subregions adjacent to the Panama Canal, as these areas contain the assemblage of birds from which the avian community on BCI is most likely derived. Subregions span the entirety of physiographic and environmental variation along the approximately 65-km long isthmus and represent a strong natural rainfall gradient from 3500 mm in the north to 1400 mm in the south, annually^34^. Multiple avian inventory methods were implemented within each subregion from 1998 to 2005, including point counts, spot mapping, and standardized area searches^37^. We removed subregions containing major cities with > 5% urban cover from our analyses (Supplementary Information, Urban Subregions).

### Species traits

To focus our study on tropical forest-associated resident birds, we removed aquatic species as well as vagrants and non-breeding migrants from the dataset. Aerially foraging birds, such as vultures, swifts, swallows, and nighthawks, were also omitted because their daily foraging ranges extend well away from BCI. For remaining species, we assigned habitat preference and residency status in the canal region based on published species accounts and extensive author experience^3,40,48,49^.

For species detected on BCI during any inventory period, we considered six categorical and two continuous attributes previously associated with extinction risk in tropical birds^7,50^ (Supplementary Information, Species Traits). Categorical traits included local abundance at time of isolation, nest type and height, primary diet, and typical foraging strata. Our two continuous species attributes were body mass and southern limit, an index of climatic tolerance. Body mass was the log-transformed mean individual weight across sexes from^51^.

We used the integer linear distance between a species southernmost Canal area occurrence and the southern entrance to the Panama Canal (“southern limit”) as a metric of dry forest tolerance. Geographic ranges of most bird species in central Panama begin in the wettest forests near the Caribbean Sea and extend some distance southward along the rainfall gradient. Species with low southern limit values reach their southern distribution boundary near the Pacific Ocean, thus they occupy a larger range in central Panama. Widely distributed species tolerate warmer, drier environments than species with higher southern limit values that are restricted to northern, wetter portions of the isthmus.

All forest birds detected on BCI were further classified as exclusively wet forest—those species only occurring in forests receiving over 2000 mm of precipitation annually, or transisthmian—birds occurring in forests across the entire precipitation gradient from wet to dry forests. To test whether the proportion of wet forest-associated species now absent from BCI is significantly different from the proportion of absent transisthmian forest birds, we performed two-proportion z-tests, applying a Yates continuity correction where necessary to account for small sample sizes.

### Environmental data

To help characterize the nature of avian community structure across the Panama Canal, we included environmental variables potentially associated with avian species distributions in the tropics^37^. These included altitude, area, degree of forest fragmentation, percent forest cover, percent urban land cover, plant species richness, and total annual precipitation (see Supplementary Table S2 for definitions and sources). We also considered forest age, which was previously found to be informative^37^. Forest age was treated as a continuous variable because categories in Rompré et al. (2007) represented a consistent, incremental series of time bins: (1) young secondary forest disturbed < 100 years ago; (2) mature secondary forest disturbed 100–500 years ago; and (3) mature primary forest not logged or cultivated for at least 500 years. Only a single set of environmental values were used for BCI regardless of year because we assume changes in the selected environmental factors on BCI over time are very small relative to the overall spatial variation in environmental factors. Temperature was not considered in analyses because daily and annual temperatures did not vary considerably across subregions.

### Statistical analyses

Analyses were performed in R 3.5.2^52^ and PC-ORD 7.1^53^. We identified important traits associated with extirpation from BCI using a combination of feature selection, logistic regression, and multiple metrics of predictor importance. Our response was whether or not a bird species is now absent from BCI. We defined “absent” birds as any species once considered a permanent resident that has gone undetected on BCI for at least a decade and has not demonstrated the capacity to re-establish breeding populations post-isolation.

#### Feature selection and regression

We used feature selection to eliminate explanatory variables not relevant to species persistence on BCI to prevent model overfitting. Feature selection was performed with the Boruta package^54^ using default settings and 1000 runs. Unlike stepwise selection procedures, Boruta identifies all relevant features instead of the minimal optimal set. This selection approach is best when the objective is to explore relationships between variables and the response, rather than produce a generalizable or predictive model^54^.

To investigate the relationships between important variables and persistence on BCI, we fitted a generalized linear model (GLM) using the stats package in R^52^. We found no evidence of multicollinearity or overdispersion in our data, so we performed regression with binomial distribution and logit link function. A logistic regression model incorporating granivores and/or arboreal foragers was not possible due to quasi-complete separation of the data (*i.e.,* certain combinations of predictor variables provided strong or perfect separation between extinct and remaining species) that was not resolved by a penalized maximum-likelihood method. Models with quasi-separation lack convergence for parameter estimation. To address this, we combined arboreal and raptorial foragers into a single category and omitted granivores from our final model. Interaction terms between diet and foraging height as well as local abundance and southern limit were considered but omitted due to lack of statistical significance.

We measured the contribution of individual species attributes to overall model fit using dominance analysis and hierarchical variance partitioning. Hierarchical variance partitioning provides a relative measure of variable contribution as a percentage of overall model performance^55^. Using the dominanceanalysis package^56^, we ranked predictor importance based on general dominance using McFadden’s pseudo-R^2^ as a measure of fit and estimated error using a bootstrapping procedure with 1000 runs. McFadden’s pseudo-R^2^ is a measure of model effectiveness conceptually and mathematically similar to R^2^ for ordinary least squares regression^57,58^. Pseudo-R^2^ scores of 0.2–0.4 should be interpreted as good model fit^59,60^. After confirming monotonicity and normality in the residual structure, we used the hier.part package^61^ to perform variance partitioning with binomial distribution and log-likelihood as the performance metric. We performed a randomization procedure with 1000 runs to determine statistical significance of variable effects.

We used a likelihood ratio F-test to evaluate the significance of the reduction in residual deviance in the fitted model relative to a null model with no parameters. Model calibration was evaluated using the le Cessie-van Houwelingen test in the rms package^62^. This test is an improved form of the Hosmer–Lemeshow goodness-of-fit test where well-fitting models show no significant differences between observed responses and predicted probabilities^63^. We also used adjusted D^2^ to measure the percentage of deviance explained by the fitted model accounting for the number of predictors and observations used^64^. Both D^2^ and pseudo-R^2^ values were calculated using the modEvA package^65^.

#### Non-parametric ordination and cluster analysis

We used non-parametric analytical techniques to holistically evaluate temporal changes in avian community composition on BCI relative to spatial community variation along the Panama Canal. Our response variable was the presence (response value of 1) or absence (response value of 0) of each bird species per sampling area. The initial dataset contained presence/absence values for 317 bird species detected at 14 non-urbanized mainland subregions, plus seven distinct BCI avian inventories and one hypothetical future BCI species list. We removed 18 bird species occurring in less than 5% of sampling units. Our secondary matrix of eight environmental variables was relativized by adjusting to standard deviates. Outlier analysis identified one sampling area, which we chose to retain (Supplementary Information, Outlier Analysis).

We performed cluster analysis to define groups of subregions with similar species composition. Our data consist of 8 current or historical inventories from BCI, but only one inventory from each mainland subregion. To demonstrate the greatest magnitude of transition between historical and current avian communities on BCI, we only used the earliest (1925–1929) and most recent (2000–2018) species inventories in cluster analysis. Cluster analysis of sampling units (subregions) by species composition involved a hierarchical agglomerative clustering strategy with Sørensen distance and the average linkage method. We used the pvclust package^66^, to perform multiscale bootstrap resampling for 10,000 cluster permutations and calculate the Approximately Unbiased (AU) probability value for each cluster. AU values of 95% or greater indicate strong statistical support for the existence of independent groups within the data^67^.

To measure the strength of differences between groups of subregions in species space identified by hierarchical cluster analysis, we used the pseudo *F-*statistic generated by permutational, nonparametric multiple analysis of variance (PerMANOVA)^68^. A hypothesis test of the *F-*ratio was not possible because testing the observed ratio in the same space that the clusters were generated will always yield a significant result. We conducted analyses using Sørensen distance with cluster assignment as a fixed group (one-way design). Because PerMANOVA is sensitive to differences in group dispersion, we tested homogeneity of cluster variance using a permutational dispersion test (PERMDISP; ^69^ that accommodates non-Euclidean distance measures. Both PerMANOVA and PERMDISP were conducted using the ‘vegan’ R package^70^.

Sampling units and bird species were ordinated in species space using nonmetric multidimensional scaling (NMDS) using Sorensen distances on random starting configurations for 250 runs on both real and randomized data. Ties in the distance matrix were not penalized. Statistical significance of the final stress was evaluated by randomization test comparing the observed final stress against that of data randomized by permuting the values within columns (species).

## Results

**Figure 2:**
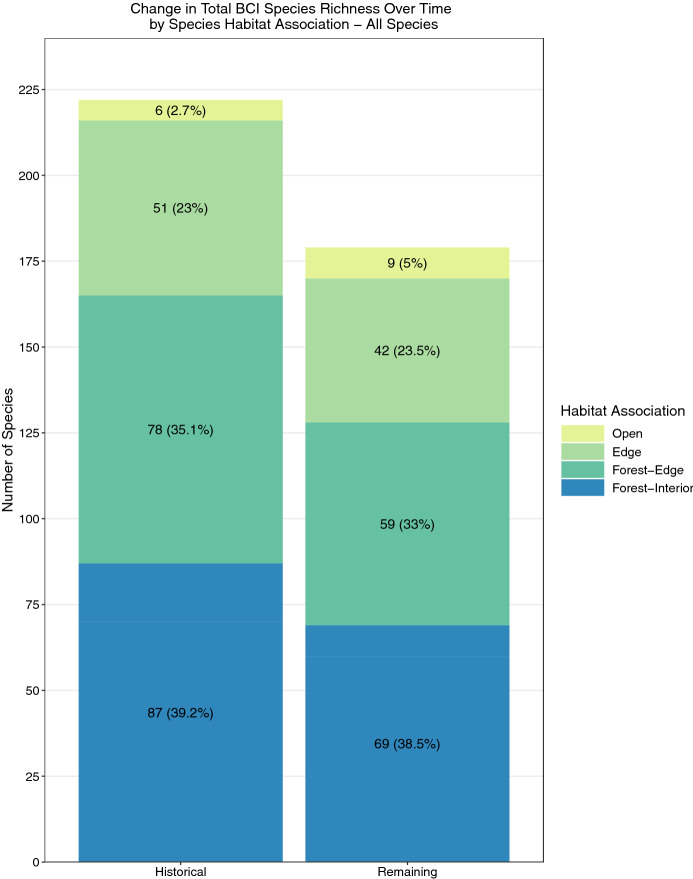
Change in BCI species richness over time by species habitat association, between the complete historical non-aerial, non-aquatic resident bird community and the remaining bird community today. “Historical” refers to pre-1951 bird community, including likely but undetected birds. The remaining bird community includes permanent residents and non-permanent species that intermittently breed on the island. Values in columns denote number of species in that habitat association group (with percentage out of total species richness for that period in parentheses).

The historical community of resident, non-aquatic, non-aerial birds on BCI before 1950 consisted of 228 species (Supplementary Table S3). Sixty-two bird species (27.2%) are now considered extinct on BCI, 37 of which are exclusively forest-associated species. Thus, 22.4% of the original 165 exclusively forest-dwelling species have been lost. Six additional species may also be extinct but are difficult to detect, largely because of their nocturnal or wide-ranging habits. Those species are omitted from further analysis. Proportionally more forest-associated species were lost than species associated with edge habitat (22.4% vs. 17.7%, respectively; Fig. 2). Species losses occurred without replacement; only one species (Great Kiskadee, *Pitangus sulphuratus*—a species of edge habitats; see Supplementary Table S3 for scientific names) colonized BCI after its isolation. Eighteen additional species were detected on BCI after 1951, primarily vagrants or “ephemeral” breeders (Supplementary Table S4). Three additional urban-associated species are experiencing range expansions in central Panama but have yet to establish stable, resident populations on BCI.

### Attributes of missing species

Three important species attributes were significantly associated with extinctions: historical local abundance; the ability to tolerate drier environments—represented by a species’ southern distributional limit across the isthmus of central Panama; and diet (Supplementary Information, Species Traits). Foraging height was identified as a tentatively important variable. A logistic regression model built using only these attributes, including foraging height, significantly reduced residual deviance from the null model (null deviance = 269.35, deviance reduction = 87.7, *p* < 0.001) and reliably characterized species absences from BCI. We found no evidence for lack of fit using the le Cessie-van Houwelingen test (z =  − 0.78, *p* = 0.43). Good model performance was confirmed with a satisfactory McFadden’s pseudo-R^2^ value (0.35). The amount of deviance accounted for by our model after adjusting for number of predictors and observations was 30%.

Dominance analysis suggests local historical abundance was the most important predictor explaining species extinctions on BCI (average increase in R^2^_M_ = 0.17, SE = 0.18) followed by southern limit (average increase in R^2^_M_ = 0.077, SE = 0.08), diet (average increase in R^2^_M_ = 0.063, SE = 0.08), and foraging height (average increase in R^2^_M_ = 0.021, SE = 0.027). Hierarchical variance partitioning indicated local historical abundance independently contributed 51.0% of total model fit, followed by southern range limit (23.5%), then diet (19.2%) and foraging height (6.3%).

Forest species are more restricted to northerly (wetter) portions of the precipitation gradient than edge and open associated birds combined (9.7 ± 10.1 km mean southern range limit vs 2.7 ± 7.1 km, respectively). Wet-forest species occur infrequently, if at all, in drier southern forests, and so exhibited a higher southern limit than transisthmian forest species (23.8 ± 7.1 km mean vs. 6.2 ± 7.4 km). Thus, wet-forest species on average span only the northernmost 63% of the 65 km-wide isthmus compared to transisthmian species, which span an average 91% of the isthmus. Differences in average southern limit between missing and remaining species are summarized in Supplementary Table S5. Species now missing from BCI have an average southern limit 8.1 km (12.5% of the isthmus) more northerly than remaining species.

A significantly greater proportion of wet-forest birds disappeared from BCI (51.6% wet vs. 15.7% transisthmian forest species absent; z_df=1_ = 16.7, *p* < 0.001; Fig. S2). This was also true when considering forest interior and forest edge species separately (forest interior z_df=1_ = 7.9, *p* = 0.002; forest edge z_df=1_ = 7.21 *p* = 0.004). We found no evidence forest interior birds lost a greater proportion of species than forest edge-associated species in either wet or transisthmian forests (wet forests z_df=1_ = 0.04, *p* = 0.58; transisthmian forests z_df=1_ = 0.05, *p* = 0.59).

### Extinction timing

Probability of species persistence significantly declined with decreasing local abundance and more restricted (northerly) range limits in central Panama, as well as terrestrial foraging and primarily insectivorous diets (Table 1). The earliest extinctions were forest-dwelling insectivores historically rare on BCI. 78.3% of disappearances in the first four decades after isolation were insectivores, suggesting insect eating birds are lost more quickly than other groups. Extinction timing was significantly correlated with southern range limit in central Panama and historical abundance. We found a moderate and significant positive correlation between extinct species’ historical abundance on BCI and decade of last observation (Spearman’s Rho = 0.34, *p* = 0.007). There was also a significant negative association between southern distributional limit and decade of last observation (Spearman’s Rho = -0.29, *p* = 0.02; Fig. S1).

**Table 1:** Results from logistic regression of local bird extinctions from BCI as a function of local historical abundance, diet category, foraging height, and southernmost Panama Canal range limit.

Parameter	β	S.E	z-value	*p*-value
Intercept	5.55	1.11	5.02	< 0.001
Southern Limit	− 0.06	0.02	− 3.51	0.001
Local Abundance—Occasional	− 2.82	1.08	− 2.61	0.0091
Local Abundance—Rare	− 3.86	1.05	− 3.66	< 0.001
Diet—Insectivore	− 1.48	0.46	− 3.24	0.001
Diet—Nectarivore	− 0.44	0.85	− 0.52	0.61
Diet—Frugivore	0.83	1.21	0.69	0.49
Diet—Raptor	− 0.06	0.60	− 0.10	0.92
Foraging Height—Understory	− 0.04	0.88	− 0.04	0.97
Foraging Height—Terrestrial	− 1.09	0.54	− 2.03	0.042

Proportionally more forest species were lost from BCI despite increasing forest cover over time. Only half of the total species extinctions (32 sp., 51.6%), but three quarters of forest-associated extinctions, occurred before 1970. Most forest-interior bird extinctions (19 sp., 82.6%) occurred before 1970, whereas most forest-edge (17 sp., 68%), edge (9 sp., 69.2%), and all open-associated species went extinct after 1970. Across all habitat association types, species lost from BCI were more restricted to the wettest end of the rainfall gradient while those remaining on BCI today are widely distributed across the entire gradient (Supplementary Information, Species Traits).

### Cluster analyses

**Figure 3:**
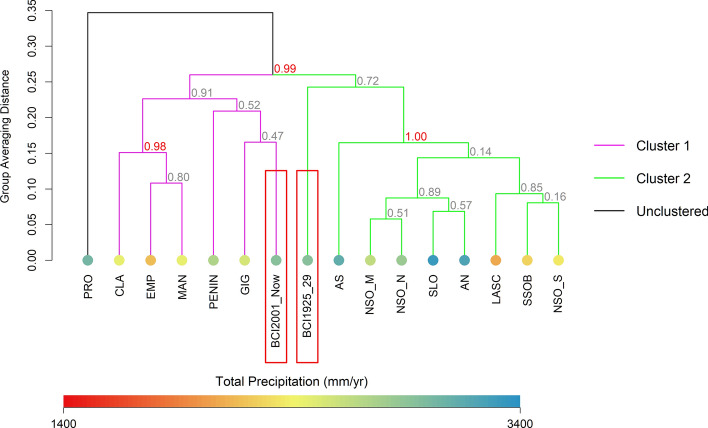
Hierarchical cluster analysis based on group average Sorensen distance. Values at nodes represent approximately unbiased (AU) probability of that cluster based on multiscale bootstrap resampling of the data for 10,000 runs. Red text indicates statistically significant clusters at *p* > 95%. Colored circles at dendrogram “leaves” correspond to total annual precipitation for that subregion. Colored lines denote independent clusters of subregions. The oldest and most recent bird inventories from BCI are indicated with red rectangles.

Hierarchical cluster analyses of 14 mainland sites and two BCI inventories (first and most recent) by 299 species revealed strong support for one cluster of mainland sites (“Cluster 1”) on the southern end of the canal and moderate support for a second cluster (“Cluster 2”) containing sites on the northeast side of the canal and west edge of Gatun Lake (Fig. 3). Supplementary Table S6 summarizes the environmental characteristics of each cluster. There was additional strong support (*p* = 0.04–0.05) for two smaller “sub-groups” within Cluster 2, differentiating northern subregions with high precipitation (> 2400 mm/yr) from drier subregions near the center of the Panama Canal (< 2250 mm/yr precipitation). Nueva Providencia (PRO) was not clustered with any other subregions and there was convincing evidence (*p* = 0.01) this site represented a distinct forest bird community. Because PRO was not a member of any cluster, we omitted this subregion from tests of heterogenous distance and dispersion among groups.

One-way PerMANOVA between clusters yielded a pseudo-*F* statistic of 8.16, suggesting differences between groups was greater than chance alone. 50.6% of the variance in the data was accounted for by differences between groups. There was no evidence that differences between groups were driven by variation in dispersion (between mainland groups F = 2.58, *p* = 0.14; including BCI communities F = 1.53, *p* = 0.27).

Cluster analyses associated the earliest inventoried BCI community with mainland sites on the northeast side of the canal and northern edge of Gatun Lake, which includes nearby mainland subregions in Soberania National Park (Fig. 3). The modern BCI bird community clustered with mainland sites on the southern end of the canal, particularly the Barro Colorado National Monument peninsula (PENIN) and Gigante (GIG; Fig. 3). These two subregions adjacent to BCI are comparatively more exposed to drying winds and more recently disturbed by anthropogenic activity and damaging storms than Soberania National Park. All bird communities associated within this cluster of southern sites, including the current BCI species assemblage, are grouped distinctly from higher precipitation northern sites.

### NMDS

Summary statistics of the initial dataset indicated a coefficient of variation (CV) of species totals of 55.8%, and CV of 22.8% for sampling unit totals. These values show low variability in marginal totals of the species matrix, such that relativizations would have little effect on the final ordination.

Our ordination with rare species removed converged on a stable 3-dimensional ordination (final stress = 7.31, final instability = 0.00) with a cumulative R^2^ of 88.7%. Axis 1 accounted for 53.8% of the variation in the data, Axis 2 accounted for 21.1%, and Axis 3 accounted for an additional 13.4% of variation. NMDS extracted stronger axes than expected by chance (*p* = 0.004). All three ordination axes exhibited correlations with at least one environmental factor (Table 2). The first axis characterized a progressive change in avian community composition with decreasing forest cover from left to right. Axis 2 was most strongly correlated with forest age and precipitation and best represented the natural rainfall gradient along the Panama Canal, with wetter subregions occurring higher on Axis 2.

**Table 2:** All Pearson *r*^2^, and Kendall (tau) correlation coefficients between environmental variables and the three-dimensional NMDS configuration of sampling units in species space.

Variable	Axis 1		Axis 2		Axis 3	
	*r*^2^	tau	*r*^2^	tau	*r*^2^	tau
Forest age	0.177	− 0.348	0.050	− 0.162	0.065	− 0.174
Altitude	0.027	− 0.014	0.001	− 0.088	0.357	0.522
Area	0.001	− 0.060	0.029	0.088	0.490	0.494
% Forest	0.274	− 0.374	0.035	− 0.152	0.269	− 0.448
% Unfragmented	0.361	− 0.416	0.007	− 0.139	0.117	− 0.416
% Urban	0.077	0.356	0.024	0.106	0.232	0.443
Plant richness	0.008	− 0.472	0.017	0.180	0.232	− 0.321
Precipitation	0.049	− 0.287	0.042	0.176	0.263	− 0.435

For definitions of environmental variables, see Supplementary Table S2.

Though most environmental factors were strongly correlated with Axis 3, the greatest correlations were with mean altitude and total area. Axis 3 generally differentiated large subregions with more topographic and habitat complexity from smaller, environmentally homogenous subregions, but did little to characterize differences between clusters or BCI bird communities over time and captured only a small amount of variation in the data. Furthermore, there was no evidence that birds occupied different areas of the ordination based on habitat association.

**Figure 4:**
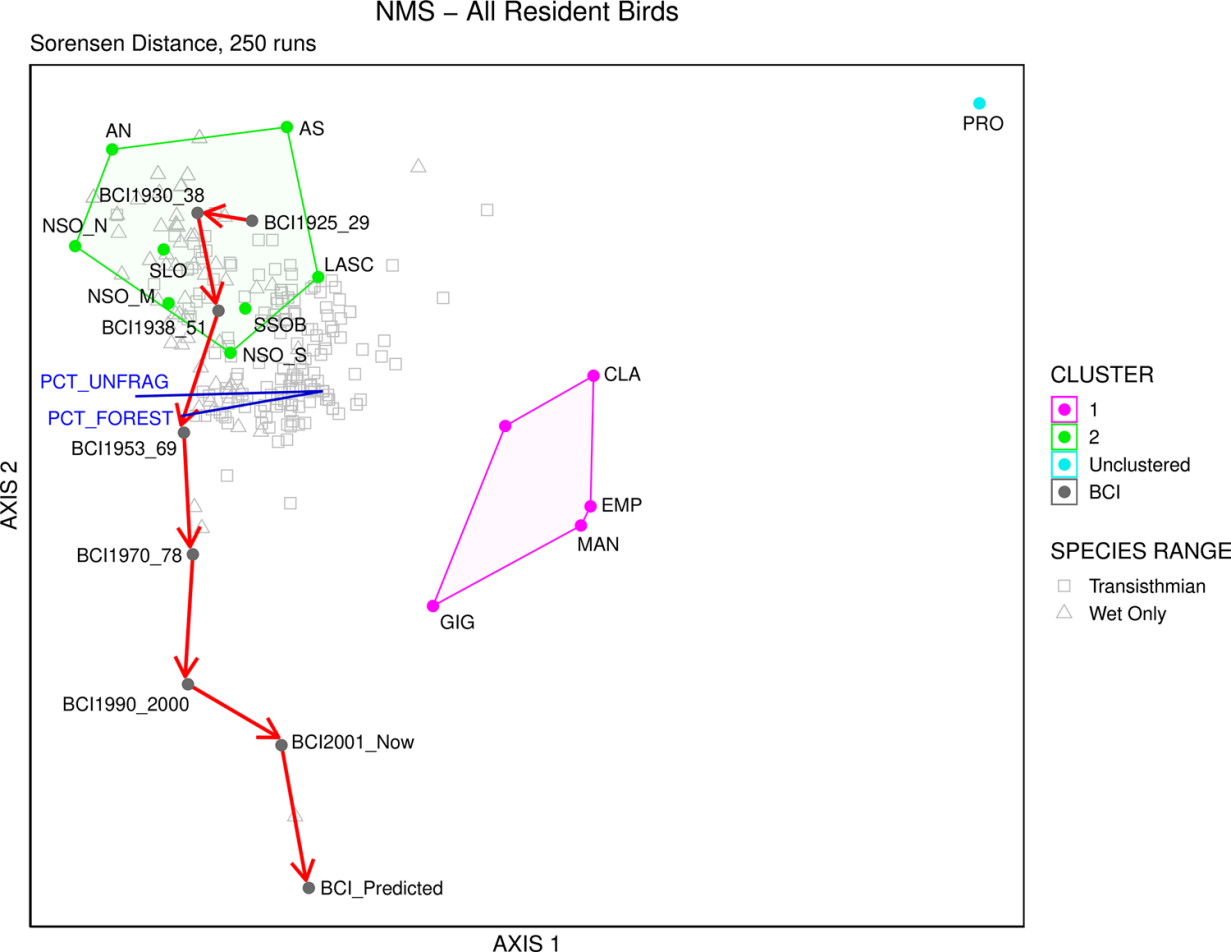
NMDS ordination of species and sampling units in species space. Subregions indicated by abbreviations, with colored dots corresponding to group membership based on hierarchical cluster analysis (Fig. 3). Numbers after BCI indicate census period. Bird species are represented by hollow gray shapes. Squares denote transisthmian birds—those occurring along the entire Panama Canal; triangles represent birds that occur in exclusively wet, Atlantic subregions. Red line is a successional vector connecting BCI censuses in chronological order. Dark blue lines and text indicate the strength and direction of significant associations between environmental variables and axes.

Ordination of non-urban subregions in species space showed BCI bird communities shifted progressively over time such that historical and modern inventories were positioned alongside different clusters of mainland sites (Fig. 4). Wet-forest birds were particularly associated with subregions having the greatest amounts of forest cover and precipitation (Fig. 4). Early BCI bird inventories (1925–1951) ordinated closely with wet-forest species and Atlantic subregions—particularly those receiving the highest amounts of precipitation. Later BCI communities ordinated progressively down the second axis, with modern and predicted BCI bird inventories alongside dry mid-isthmus and Pacific slope subregions.

## Discussion

Despite possessing several factors thought to minimize loss of biodiversity in habitat remnants: large patch size, a surrounding habitat matrix resistant to exotic invasion, and effective protection from human disturbance for nearly a century, Barro Colorado Island has lost nearly a quarter of its strictly forest-associated bird community. The historic and present-day BCI bird communities are dissimilar and ordinate most closely with bird communities in wet versus drier mainland subregions, respectively. The “unprecedented record of natural extinctions”^10^ from BCI reveals three primary factors driving species loss: low initial abundances, dietary specialization on primarily terrestrial insects, and sensitivity to forest moisture conditions. Extirpated species tend disproportionately to be associated with wet forests in the region. Moisture conditions, largely driven by precipitation across the transisthmian rainfall gradient, are an important driver of community characteristics. Forest-dwelling species of the interior and the edges of tall forest (i.e., species preferring gaps and the canopy of forest) have declined in parallel. All species remain present in comparably-sized mainland forests and are absent from the other Gatun islands^71^, indicating the losses from BCI are influenced by its isolation from mainland forests.

### Effects of abundance, diet, and climatic tolerance

Local abundance was the most important variable explaining species losses; species with lower abundance at the time of isolation were generally the first to become extinct. Small populations within isolated fragments may lack the density necessary to support viable populations over long time periods. Large populations are buffered against stochastic fluctuations which carry small populations below the minimum abundance threshold necessary for persistence^72^. Though almost all birds lost from BCI maintain populations in adjacent mainland fragments, some less than 250 m away, physical and behavioral limitations—especially among terrestrial birds—likely prevent dispersal events across the lake^8,9,39,73^. Without the ability to replenish isolated populations from outside sources, species with small enough initial populations eventually become extinct.

Species that specialize on terrestrial insects and occasional small vertebrates (hereafter terrestrial insectivores) were significantly less likely to persist. Stouffer and Bierregaard^20^ postulated that alterations of ground-level vegetation structure in small forest fragments, caused by increased treefall and changes in leaf litter composition, could reduce the ability of terrestrial insectivorous birds to locate prey. Though BCI is considered large enough to buffer against many of these negative effects observed in smaller patches, the wind-exposed edges of BCI’s peninsulas still experience a considerable exposure effect^74^. Tropical forest insectivores also occur at lower densities than other groups^49^. Wolda^75^ found insect populations in Panamanian forests fluctuate widely over time. Insectivores with naturally low abundances may be particularly sensitive to periodic fluctuations in insect populations^75^. However, data on long-term insect population trends for the tropics remain scarce.

Ant-followers are a specialist insectivorous guild that feeds on terrestrial arthropods flushed by army ant swarms. Obligate ant-following birds are thought to be particularly vulnerable to fragmentation due to low population densities and extreme reluctance to cross open areas between forests^6,20,38^. Two of the three obligate ant-followers once found on BCI are now absent. The two missing ant-followers were historically less abundant and possess more northerly central Panama range limits than the species that remains. It is unlikely these species went extinct on BCI due to lack of ant colonies given stable numbers of ant swarms over time^39,76^. Rather, these losses appear consistent with our findings that numerically rare terrestrial insectivores near the limits of their distributions and/or climatic tolerance are among the most sensitive to isolation.

Fine-scale habitat specialization can be a strong predictor of avian sensitivity to anthropogenic change along habitat gradients^77^. We found a species’ climatic tolerance, indexed by their southernmost occurrence in the Canal area relative to the driest part of the central Panama isthmus, was significantly associated with extinction risk. As species limited to wetter, northern forests in central Panama disappeared from BCI over time, the average distributional extent of birds still present on BCI increased. The bird community on BCI is increasingly composed of regionally widespread species.

Tropical species that live in less seasonal environments possess narrower physiological tolerances than those living in situations with widely fluctuating conditions^19,78^. Persistence may be influenced by the ability to make seasonal movements in search of suitable climatic conditions during periodic dry spells^79^. Like many hilltops transformed to habitat fragments after reservoir creation, BCI has insufficient refugia in its forest interior and its surrounding matrix of water effectively eliminates movements to or from the island for dispersal-limited birds^8,73^. Yet, isolation by water does not limit generalizability of these consequences of isolation because experimental evidence for dispersal limitation has documented similar behaviors of birds in fragments surrounded by pasture^20,80^.

Some losses among edge or canopy-associated species are attributable to maturation of early and secondary growth as a result of vegetation succession^10,38^. However, our observation that forest birds (both forest-interior and forest-edge) experienced greater proportional declines than edge-associated species suggests loss of young forest was not exclusively responsible for the observed extinctions. Though we did not find a relationship between habitat affiliation and extinction risk, there is evidence forest birds are more sensitive to fragmentation. The majority of extinctions, and all extinctions in the first 30 years, were species of the forest interior. If currently declining populations of species with traits similar to those species already lost continue to dwindle, we expect about 12 additional species to be lost in the next two decades and we anticipate even greater alignment of the BCI community with drier-forest communities.

### Evidence for fragmentation-associated loss of moist refugia

Our study provides comprehensive evidence reduced access to humid forest conditions contributes to the structure of an isolated tropical bird community. After a century of species loss disproportionately affecting humid-forest specialists, our results indicate the BCI bird community has shifted in composition over time, progressing from initially being similar to nearby wet-forest bird communities to now being most similar to communities in drier and more disturbed forests. The loss of terrestrial insectivores resembles community shifts observed in undisturbed Amazonian forests that may be associated with changes in rainfall^81,82^. However, the observed shift in avian community composition on BCI towards drier habitat assemblages did not correspond with a reduction in total annual precipitation or soil moisture.

This community shift without corresponding change in annual rainfall may be explained in part by an increase in the proportion of marginal or unsuitable habitat for wet-forest birds as a consequence of fragmentation and isolation. BCI’s many peninsulas experience a strong desiccating exposure effect from winds across Gatun Lake^74^; even the core island interior contains little permanent water compared to nearby forest patches of comparable size. Vegetation changes on BCI, where moisture conditions are an important factor determining long-term tree species composition^83^, are consistent with an overall drier environment. Tree mortality rates, particularly among species associated with moist slopes, appear to be rising recently despite stable temperatures^84^. Areas on BCI that accumulate water seasonally are desiccating and becoming less distinct from surrounding vegetation^83^. Our study suggests changes in species composition associated with exposure and loss of seasonal water sources in the forest interior extends to birds as well.

Environmental consequences of fragmentation may interact with a pronounced annual dry season that varies in length and severity. Though total annual rainfall remains near its century-long average, there is evidence of increasing rainfall variability recently, with more frequent extreme wet and dry periods across the region^85^. Karr^11^ hypothesized the occasional extreme dry period is important for tropical species losses, where even routine dry seasons may critically stress species that rely on moist refuges, perhaps more so if their population sizes are already small. Drier tropical forests have lower densities of arthropod decomposers^86^ and dry periods limit the above-ground activity of terrestrial insects^87,88^. Experimental evidence shows the distribution and density of forest-floor arthropods on BCI is strongly associated with leaf litter moisture content during the dry season^89^. Length of Panamanian dry season is negatively correlated with avian demographic rates^90^ and is likely a limiting factor for animal populations on BCI. Reductions in terrestrial arthropod activity and subsequent trophic consequences of more frequent extended drought could help explain the loss of terrestrial insectivores in particular from BCI. Periods of increased extinction appeared to follow periods of reduced rainfall and steep avian declines in 1960s and 70s corresponded with a prolonged period of below-average precipitation^36^.

Affinity with drier climates is associated with avian persistence in tropical agricultural landscapes, where agricultural conversion changes the regional ecosystem and vegetation structure into ones more characteristic of drier biomes^91,92^. However, many aspects of BCI’s floristic structure still resemble a typical wet forest. Our results suggest subtle changes in habitat conditions associated with isolation have disproportionate effects on the avian community, although the mechanistic explanations at the organismal level still need to be identified.

### Additional considerations

Even though the BCI forest has remained undisturbed by human activity for nearly a century, the bird community is now more similar to a set of less species rich communities in drier, disturbed forests in the region. This observed shift in community assemblage on BCI over time is made without equivalent temporal data on compositional change in the regional avifauna. The century-long history of bird surveys on BCI is unique. It is possible mainland bird communities along the Canal have also changed, which would not be depicted by our ordination. Anecdotal evidence from Soberania National Park suggests some minor compositional shifts have occurred since the 1970s. However, those few losses from Soberania are limited to species even more range-restricted to the wettest forests in central Panama. More recent inventories of other islands in Gatun Lake, all smaller than BCI, reveal an absence of wet-forest species as well^71,93^.

BCI is also probably the only subregion that has gained tall forest coverage over time. Most common forms of anthropogenic disturbance on the mainland, including forest removal, agricultural conversion, and urbanization, do not affect Barro Colorado Island. Shifts between historical and modern bird communities in Soberania appear to be along the horizontal axis of the ordination defined by forest cover and fragmentation, not in the same direction of change experienced by BCI.

## Conclusion

Barro Colorado Island, lacking permanent streams in the forest interior and with a large proportion of its perimeter exposed to edge effects, appears to be a generally inhospitable habitat for wet-forest birds that rely on moist refugia to withstand seasonal and periodic dry spells. These birds, declining in unsuitable remnant habitat and unable to replenish their populations from nearby forests, slowly perish at a rate influenced by their population size at the time of isolation and their dietary preferences.

Our results from this large land-bridge island reveal a cryptic and overlooked extinction pathway that may also be relevant for bird populations in other forest remnants where the surrounding vegetation is never permitted to regrow. Hilltop forest fragments are common throughout the tropics and often lack permanent streams and the microhabitat regimes associated with them. This reduced set of environmental conditions, combined with isolation, predisposes a non-random subset of moisture-sensitive and dispersal-limited species to be more prone to extirpation. Increasing dry season length and more frequent intense droughts predicted by many climate models^85,94^ may drive further species losses on BCI. The proposition that simply protecting rainforests will maintain their diversity over the long-term is increasingly unsupported given recent findings of bird declines in vast undisturbed Amazonian forests as well as shifts in tree community composition^81,82,95^. Ongoing BCI species losses, even in this large remnant protected from human disturbance for over a century, reveal important influences of initial population size and specialization interacting synergistically with subtle effects of habitat isolation to slowly erode biodiversity.

## Supplementary Information

Supplementary Information 1.
