# Supplementary material for: Erosion of tropical bird diversity over a century is influenced by abundance, diet and subtle climatic tolerances
Source: Sci Rep. 2021 May 11;11:10045. doi: 10.1038/s41598-021-89496-7 (PMC8113325; doi:10.1038/s41598-021-89496-7)
Supplement: Supplementary file 1 — Supplementary Information 1. [file 41598_2021_89496_MOESM1_ESM.pdf]

Supplementary Information

Erosion of tropical bird diversity over a century is influenced by abundance, diet and subtle climatic tolerances

Jenna R. Curtis[1], W. Douglas Robinson[1], Ghislain Rompré[1], Randall P. Moore[1] & Bruce McCune[2]

[1] Department of Fisheries, Wildlife and Conservation Sciences, Oregon State University, 104 Nash Hall, Corvallis, OR 97331;

[2] Department of Botany and Plant Pathology, Oregon State University, 2082 Cordley Hall, Corvallis, OR 97331

Species Traits

For resident non-aquatic bird species in this study, we assigned preference for one of four habitat categories: open areas with little to no woody vegetative cover; edge habitat at the boundary of low, woody vegetation; the outer margins of forest of any age (forest edge); and interior of forest of any age. Residency status was classified as permanent resident or breeding migrant (*i.e.,* seasonally occurring nesting species).

For species detected on BCI during any inventory period, we considered an additional six categorical and two continuous attributes previously associated with extinction risk in tropical birds [1,2]. Abundance on BCI was categorized based on estimated total island-wide population at the time of isolation [3]: common (> 100 individuals); occasional (10-100 individuals); or rare (<10 individuals). Regional abundance was considered but ultimately omitted due to strong correlations with several other variables.

Artificial nest experiments in tropical forest fragments, including on BCI, posited that the absence of large predators and subsequent increase in middle-sized nest predator abundance was responsible for avian extinctions, particularly among ground-dwelling species [4–6]. However, recent experimental and observational evidence suggests artificial nest studies lack sufficient external validity [7–9] and traditional theories about the identities of primary nest predators in tropical forests appear misinformed [10–12].

To further evaluate the extent that nest attributes contribute to fragmentation sensitivity despite the lack of observational evidence in support of increased depredation rates by mesopredators, we included typical nest height and type as potential predictors of extinction risk on BCI. We assigned one of four possible categories for a species' typical nesting height: ground (<1m); understory (1-5m); mid-story (>5m but below the canopy); or canopy (the top level of vegetation regardless of height). We used three categories for nest type: open cup (bowl, platform, or scrape); enclosed, roofed nests with a single entrance (e.g. pendulum, pyriform, or pouch); or cavity nests in trees, burrows, or termite mounds. Obligate brood parasites were assigned the nest attributes of their most common hosts.

We established six dietary guilds according to a species' primary food source [13]: carnivores (vertebrates, snails, carrion, and occasional large arthropods); frugivores (fruits of any size); granivores (seeds and nuts); insectivores (insects, arthropods, and occasional small vertebrates); nectarivores (flower nectar); and omnivores (generalists which consume food from more than one category). We compiled four categories for the typical height at which a species searches for food: terrestrial (<1m); understory (greater than >1m but below midstory); arboreal (uppermost layers of vegetation, including midstory and canopy, regardless of height); and raptorial hunters that pursue prey across all forest strata. Aerially foraging birds, such as vultures, swifts, swallows, and nighthawks, were omitted because their daily foraging ranges extend well away from BCI.

Our two continuous species attributes were body mass and southern limit, an index of climatic tolerance. Body mass was the log-transformed mean individual weight across sexes from [14]. We used a species' southern distributional limit in the Canal area - the integer linear distance between a species' southernmost occurrence on the Panama isthmus and the Pacific entrance to the Panama Canal - as an index of climatic tolerance.

Outlier Analysis

Outlier analysis using Sørensen distance identified one sampling area, Nueva Providencia (PRO), with a distance value more than 2 standard deviations greater than the mean distance between sites (SD = 3.5). PRO is isolated on the northeastern edge of Gatun Lake and not adjacent to any other evaluated subregion (Figure 1). This subregion contained low avian species richness relative to the average for non-urban mainland areas (120 species vs. mean richness of 198) and exhibited peripheral placement with repulsion on at least one axis in preliminary ordinations. PRO did not possess extreme or unusual values for any environmental variables. We believe this subregion was identified as an outlier because it was the only sampling unit on the northeastern side of the Canal area and, without nearby avian communities of similar species composition, was not as easily ordinated in the context of the other subregions. Additional data from this general region would likely reduce the significance of PRO as an outlier. This sampling unit represents an area for which we have no other avian inventory data from

predominantly forested habitat. Because we considered PRO to be within our target population, its bird inventory was as complete as other subregion inventories [15], and we have no reason to doubt the accuracy of its data, we retained this subregion for analysis.

Likely Species

Several difficult to identify or detect species, including nocturnal birds and small upper-canopy passerines, were missing from earlier datasets, likely due to observer inexperience with tropical bird vocalizations [16] or lack of access to all parts of the island. To evaluate the effect of these common but poorly detected species (AKA “likely species”), we repeated ordination procedures on a reduced species matrix where 21 species we deemed were likely present during historical surveys but not reported were removed (Table S1). To quantify the effect of likely species on the ordination, we compared NMDS ordination scores between the primary and reduced species datasets using Mantel’s asymptotic approximation method with a randomization test for 999 runs. This evaluates the null hypothesis of no correlation between distance matrices for the same sampling units. [17]. The standardized Mantel statistic (r) ranges from 0 to 1, with larger values representing higher correspondence between two ordinations. We calculated the percentage redundancy of the ordinations as r^2 multiplied by 100. We found strong agreement between ordinations including and omitting these species (r = 0.991). We conclude missed detections resulting from historical observer inexperience or unfamiliarity with a few tropical bird sounds did not appreciably influence NMDS results.

Urban Subregions

BCI has <0.02% urban land cover surrounding a scientific research station, and is otherwise forest, disturbed only by natural wind events. Mainland subregions along the Panama Canal range from <1% to over 97% urban land cover. Urbanized areas in this region have outsized effects on habitat and avian community composition. Sampling units with one or more large cities within their boundaries, despite having as little as 5% total urban cover, generally contained depauperate bird communities in small, remnant patches of degraded forest amidst large zones of species-poor anthropogenically altered habitat [15]. Our objective was to evaluate changes in the BCI bird community relative to comparable mainland forest habitats and bird communities. Because even lightly urbanized areas in central Panama appear to experience different structuring mechanisms than wholly forested areas, we removed subregions containing major cities with >5% urban cover from our analyses. Selective logging, fire, and other forms of disturbance common elsewhere in the tropics are rare within the forests of the Canal area subregions retained for analyses.

References for Supplementary Information

1. Henle, K., Davies, K. F., Kleyer, M., Margules, C. & Settele, J. Predictors of species sensitivity to fragmentation. *Biodiversity & Conservation* **13**, 207–251 (2004).
2. Sodhi, N. S., Liow, L. H. & Bazzaz, F. A. Avian Extinctions from Tropical and Subtropical Forests. *Annu. Rev. Ecol. Evol. Syst.* **35**, 323–345 (2004).
3. Chapman, F. M. *My tropical air castle*. (D. Appleton and Co., 1929).
4. Loiselle, B. A. & Hoppes, W. G. Nest predation in insular and mainland lowland rainforest in Panama. *The Condor* **85**, 93–95 (1983).
5. Sieving, K. E. Nest predation and differential insular extinction among selected forest birds of central Panama. *Ecology* **73**, 2310–2328 (1992).
6. Gibbs, J. P. Avian nest predation in tropical wet forest: an experimental study. *Oikos* 155–161 (1991).

7. Robinson, W. D., Robinson, T. R., Robinson, S. K. & Brawn, J. D. Nesting success of understory forest birds in central Panama. *Journal of Avian Biology* **31**, 151–164 (2000).
8. Moore, R. P. & Robinson, W. D. Artificial bird nests, external validity, and bias in ecological field studies. *Ecology* **85**, 1562–1567 (2004).
9. Robinson, W. D., Styrsky, J. N. & Brawn, J. D. Are artificial bird nests effective surrogates for estimating predation on real bird nests? A test with tropical birds. *The Auk* **122**, 843–852 (2005).
10. Robinson, W. D. & Robinson, T. R. Observations of predation events at bird nests in central Panama. *Journal of Field Ornithology* **72**, 43–48 (2001).
11. Robinson, W. D., Rompré, G. & Robinson, T. R. Videography of Panama bird nests shows snakes are principal predators. *Ornitologia Neotropical* (2005).
12. Rompré, G. & Robinson, W. D. Predation, nest attendance, and long incubation periods of two Neotropical antbirds. *Ecotropica* **14**, 81–87 (2008).
13. Wilman, H. *et al.* EltonTraits 1.0: Species-level foraging attributes of the world's birds and mammals. *Ecology* **95**, 2027–2027 (2014).
14. Dunning Jr, J. B. *CRC handbook of avian body masses*. (CRC press, 2007).
15. Rompré, G., Douglas Robinson, W., Desrochers, A. & Angehr, G. Environmental correlates of avian diversity in lowland Panama rain forests: Environmental correlates of avian diversity. *Journal of Biogeography* **34**, 802–815 (2007).
16. Willis, E. O. & Eisenmann, E. A revised list of birds of Barro Colorado Island, Panamá. *Smithsonian Contributions to Zoology* 1–30 (1979) doi:10.5479/si.00810282.291.
17. Mantel, N. The detection of disease clustering and a generalized regression approach. *Cancer research* **27**, 209–220 (1967).
18. Pyke, C. R., Condit, R., Aguilar, S. & Lao, S. Floristic composition across a climatic gradient in a neotropical lowland forest. *Journal of vegetation science* **12**, 553–566 (2001).
19. ANAM. *Informe Final de Resultados de la Cobertura Boscosa y uso del Suelo de la Republica de Panamá: 1992–2000*. (La Autoridad Nacional para el Ambiente (ANAM) y The International Tropical Timber Organization Panamá, 2003).

20. Panama Canal Authority (ACP), Meteorology and Hydrology Branch. http://www.pancanal.com (2016).

21. Condit, R. *Tropical forest census plots: methods and results from Barro Colorado Island, Panama and a comparison with other plots*. (Springer Science & Business Media, 1998).

22. Pérez, R. A. *et al.* Tree species composition and diversity in the Upper Chagres River Basin, Panama. *The Rio Chagres, Panama: A Multidisciplinary Profile of a Tropical Watershed Series* (2005).

23. Santiago, L. S. & Mulkey, S. S. Leaf productivity along a precipitation gradient in lowland Panama: patterns from leaf to ecosystem. *Trees* **19**, 349–356 (2005).

Supplementary Tables and Figures

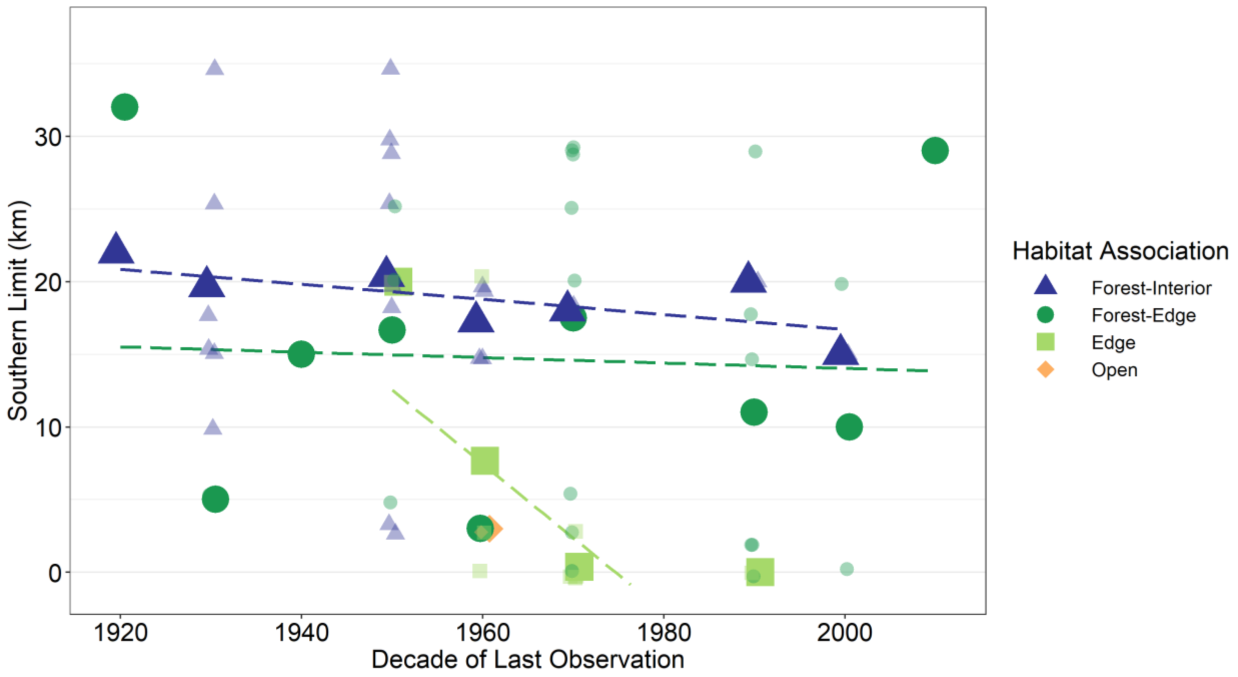

Figure S1. Southern Panama Canal area range limits for extinct species on BCI. Grouped by date of last reported observation and habitat association, with best-fit linear regression lines between southern limit and decade of extinction (dashed lines). Small shapes are individual extinctions, large shapes represent individual decade means for each habitat type.

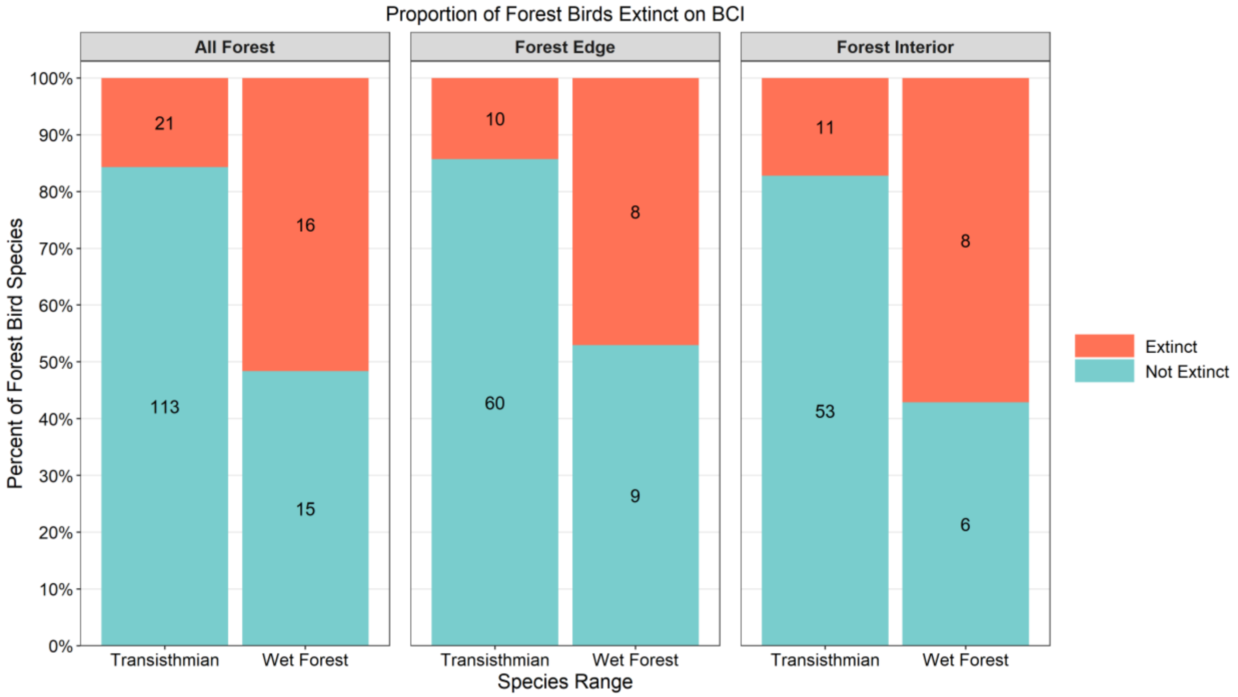

Figure S2. Proportion of transisthmian and wet-forest birds extinct on BCI across all forest-associated species as well as broken down by core forest interior and forest edge association. Transisthmian birds occur in subregions along the entire Canal area, while wet-forest species occur exclusively in forests with >2000mm precipitation annually. Extinct birds are any species considered to have once been a permanent breeding resident that has gone undetected for a least a decade and has not demonstrated the capacity to re-establish breeding populations on the island post-isolation.

Table S1. Common names of “likely species” – difficult to detect species missing from species inventories, likely due to observer unfamiliarity with these species, lack of nocturnal surveying, or inability to access all parts of Barro Colorado Island. See Table S3 for corresponding scientific names.

**Common***
Short-tailed Nighthawk
Choco Screech-Owl*
Black-and-white Owl*
Collared Forest-Falcon
Brown-capped Tyrannulet
Yellow-crowned Tyrannulet
Forest Elaenia*
Mistletoe Tyrannulet
Black-capped Pygmy-Tyrant
Sulphur-rumped Tanager
Scarlet-thighed Dacnis
Tiny Hawk*
Long-tailed Tyrant*
Scrub Greenlet*
White-vented Euphonia*
Dusky-faced Tanager*
Tropical Pewee*
White-headed Wren*
Buff-rumped Warbler*
Black-headed Saltator*
Moustached Antwren

***** Denotes species likely present on BCI at the time of isolation but not detected before 1950.

Table S2. Environmental factors used to characterize avian community structure along the Panama Canal corridor. Full descriptions of these environmental factors can be found in Rompré et al. [15].

**Variable**	**Description**	**Source**
Forest Age	Categorical. Represents dominant relative forest age (1=secondary; 2=mature secondary; 3=primary mature forests) estimated using ANAM (2003) criteria for species composition and disturbance history.	[18]
Altitude	Maximum altitude per subregion in meters above sea level.	[15]
Area	Total subregion area in km^2	[15]
% Forest	Proportional forest cover, calculated by dividing forested area by total area in each subregion.	[15]
% Unfragmented	Degree of fragmentation, represented by percent total forest area included in 1 or 2 largest fragments within subregion.	[15]
% Urban	Proportion urban cover, calculated by dividing urban area by total area in each subregion.	[19,20]
Plant Richness	Plant and tree species richness within 1 ha plots. Plant data provided by Pyke et al. (2001). Trees defined as woody plants >10cm dbh. Kriging used to interpolate geographic plant richness for each subregion centroid [15].	[18,21,22]
Precipitation	Mean annual precipitation in mm for subregion, obtained from both ACP and atlases. For subregions without precipitation data, values obtained by interpolation from isohyets available for that period [15].	[18,20,21,23]

Table S3. Common and scientific name, extinction status, and select species attributes for all birds observed on Barro Colorado Island, Panama since 1925.

Common Name	Scientific Name	Missing*	Habitat	BCI Historical Abundance	Diet	Foraging Height	Southern Limit (km)	Last Decade†
Great Tinamou	*Tinamus major*	No	forest - interior	common	omnivore	terrestrial	2	-
Little Tinamou	*Crypturellus soui*	No	edge	rare	omnivore	terrestrial	0	Predicted
Gray-headed Chachalaca	*Ortalis cinereiceps*	Yes	edge	rare	omnivore	arboreal	0	1970
Crested Guan	*Penelope purpurascens*	No	forest - interior	common	omnivore	arboreal	22	-
Great Curassow	*Crax rubra*	Yes	forest - interior	rare	omnivore	terrestrial	22	1920
Marbled Wood-Quail	*Odontophorus gujanensis*	Yes	forest - interior	rare	omnivore	terrestrial	20	1950
Pale-vented Pigeon	*Patagioenas cayennensis*	No	forest - edge	common	frugivore	arboreal	25	-
Scaled Pigeon	*Patagioenas speciosa*	No	forest - edge	common	frugivore	arboreal	0	-
Short-billed Pigeon	*Patagioenas nigrirostris*	No	forest - edge	common	frugivore	arboreal	22	-
Plain-breasted Ground-Dove	*Columbina minuta*	No	open	rare	omnivore	arboreal	0	-
Ruddy Ground-Dove	*Columbina talpacoti*	No	edge	occasional	omnivore	arboreal	0	-
Blue Ground-Dove	*Claravis pretiosa*	No	forest - edge	rare	omnivore	arboreal	0	-
Ruddy Quail-Dove	*Geotrygon montana*	No	forest - interior	common	frugivore	terrestrial	10	-
Violaceous Quail-Dove	*Geotrygon violacea*	No	forest - interior	rare	frugivore	terrestrial	17	Predicted
White-tipped Dove	*Leptotila verreauxi*	No	edge	occasional	frugivore	terrestrial	0	-
Gray-chested Dove	*Leptotila cassinii*	No	forest - interior	common	frugivore	terrestrial	0	-
Squirrel Cuckoo	*Piaya cayana*	No	forest - edge	common	insectivore	arboreal	0	-

Striped Cuckoo	*Tapera naevia*	No	edge	rare	insectivore	arboreal	30	-
Pheasant Cuckoo	*Dromococcyx phasianellus*	No	forest - interior	rare	insectivore	terrestrial	2	Predicted
Rufous-vented Ground-Cuckoo	*Neomorphus geoffroyi*	Yes	forest - interior	rare	insectivore	terrestrial	15	1930
Greater Ani	*Crotophaga major*	No	forest - edge	common	insectivore	arboreal	0	-
Smooth-billed Ani	*Crotophaga ani*	Yes	edge	rare	insectivore	arboreal	0	1970
Common Pauraque	*Nyctidromus albicollis*	No	edge	rare	insectivore	terrestrial	0	-
Great Potoo	*Nyctibius grandis*	Yes*	forest - edge	rare	insectivore	arboreal	5	2010
Common Potoo	*Nyctibius griseus*	Yes*	forest - edge	rare	insectivore	arboreal	0	2010
White-necked Jacobin	*Florisuga mellivora*	No	forest - edge	common	nectarivore	arboreal	0	-
Rufous-breasted Hermit	*Glaucis hirsutus*	No	forest - edge	rare	nectarivore	arboreal	25	-
Band-tailed Barbthroat	*Threnetes ruckeri*	No	forest - interior	rare	nectarivore	arboreal	5	-
Green Hermit	*Phaethornis guy*	No	forest - interior	rare	nectarivore	arboreal	29	-
Long-billed Hermit	*Phaethornis longirostris*	No	forest - interior	common	nectarivore	arboreal	0	-
Stripe-throated Hermit	*Phaethornis striigularis*	No	forest - interior	common	nectarivore	arboreal	0	-
Purple-crowned Fairy	*Heliothryx barroti*	No	forest - edge	rare	nectarivore	arboreal	15	-
Black-throated Mango	*Anthracothorax nigricollis*	Yes*	edge	common	nectarivore	arboreal	0	1970
Rufous-crested Coquette	*Lophornis delattrei*	Yes	forest - edge	rare	nectarivore	arboreal	15	1990
Long-billed Starthroat	*Heliomaster longirostris*	Yes	edge	rare	nectarivore	arboreal	0	1970
Garden Emerald	*Chlorostilbon assimilis*	Yes	edge	common	nectarivore	arboreal	0	1970

White-vented Plumeleteer	*Chalybura buffonii*	No	forest - interior	rare	nectarivore	arboreal	0	-
Crowned Woodnymph	*Thalurania colombica*	No	forest - interior	common	nectarivore	arboreal	0	-
Blue-chested Hummingbird	*Amazilia amabilis*	No	forest - edge	common	nectarivore	arboreal	5	-
Snowy-bellied Hummingbird	*Amazilia edward*	No	edge	occasional	nectarivore	arboreal	0	-
Rufous-tailed Hummingbird	*Amazilia tzacatl*	No	edge	common	nectarivore	arboreal	0	-
Sapphire-throated Hummingbird	*Lepidopyga coeruleogularis*	No	edge	occasional	nectarivore	arboreal	0	-
Violet-bellied Hummingbird	*Juliamyia julie*	No	forest - interior	common	nectarivore	arboreal	0	-
Gray-headed Kite	*Leptodon cayanensis*	No	forest - edge	occasional	raptor	raptorial	2	-
Hook-billed Kite	*Chondrohierax uncinatus*	No	forest - edge	rare	raptor	raptorial	15	-
Pearl Kite	*Gampsonyx swainsonii*	No	open	rare	raptor	raptorial	0	-
White-tailed Kite	*Elanus leucurus*	No	open	rare	raptor	raptorial	0	-
Double-toothed Kite	*Harpagus bidentatus*	No	forest - interior	occasional	raptor	raptorial	2	-
Tiny Hawk	*Accipiter superciliosus*	Yes	forest - edge	rare	raptor	raptorial	29	1970
Bicolored Hawk	*Accipiter bicolor*	No	forest - interior	rare	raptor	raptorial	29	-
Crane Hawk	*Geranospiza caerulescens*	No	forest - interior	rare	raptor	raptorial	12	-
Common Black Hawk	*Buteogallus anthracinus*	No	edge	rare	raptor	raptorial	0	-
Great Black Hawk	*Buteogallus urubitinga*	No	edge	rare	raptor	raptorial	0	-
Roadside Hawk	*Rupornis magnirostris*	Yes	edge	rare	raptor	raptorial	0	1970

White Hawk	*Pseudastur albicollis*	No	forest - edge	rare	raptor	raptorial	2	-
Semiplumbeous Hawk	*Leucopternis semiplumbeus*	No	forest - interior	occasional	raptor	raptorial	12	-
Gray-lined Hawk	*Buteo nitidus*	No	forest - edge	rare	raptor	raptorial	0	-
Crested Eagle	*Morphnus guianensis*	Yes	forest - edge	rare	raptor	raptorial	29	1970
Harpy Eagle	*Harpia harpyja*	Yes	forest - interior	rare	raptor	raptorial	29	1950
Black Hawk-Eagle	*Spizaetus tyrannus*	No	forest - edge	occasional	raptor	raptorial	10	-
Ornate Hawk-Eagle	*Spizaetus ornatus*	Yes	forest - edge	rare	raptor	raptorial	29	1970
Black-and-white Hawk-Eagle	*Spizaetus melanoleucus*	No	forest - edge	rare	raptor	raptorial	25	-
Tropical Screech-Owl	*Megascops choliba*	Yes	edge	rare	raptor	raptorial	20	1950
Choco Screech-Owl	*Megascops centralis*	No	forest - interior	occasional	raptor	raptorial	15	-
Crested Owl	*Lophostrix cristata*	Yes*	forest - interior	occasional	raptor	raptorial	20	2000
Spectacled Owl	*Pulsatrix perspicillata*	No	forest - interior	occasional	raptor	raptorial	10	-
Mottled Owl	*Ciccaba virgata*	Yes*	forest - edge	occasional	raptor	raptorial	0	2010
Black-and-white Owl	*Ciccaba nigrolineata*	No	forest - edge	occasional	raptor	raptorial	5	-
Slaty-tailed Trogon	*Trogon massena*	No	forest - interior	common	omnivore	arboreal	0	-
Black-tailed Trogon	*Trogon melanurus*	No	forest - interior	rare	omnivore	arboreal	15	Predicted
White-tailed Trogon	*Trogon chionurus*	No	forest - interior	occasional	omnivore	arboreal	15	-
Gartered Trogon	*Trogon caligatus*	No	forest - interior	common	omnivore	arboreal	0	-
Black-throated Trogon	*Trogon rufus*	No	forest - interior	common	omnivore	arboreal	5	-
Whooping Motmot	*Momotus subrufescens*	No	forest - edge	occasional	omnivore	arboreal	0	-
Rufous Motmot	*Baryphthengus martii*	No	forest - interior	common	omnivore	arboreal	5	-

Broad-billed Motmot	*Electron platyrhynchum*	No	forest - interior	occasional	omnivore	arboreal	5	-
White-necked Puffbird	*Notharchus hyperrhynchus*	Yes	forest - edge	rare	insectivore	arboreal	5	1970
Black-breasted Puffbird	*Notharchus pectoralis*	No	forest - edge	common	insectivore	arboreal	15	-
Pied Puffbird	*Notharchus tectus*	Yes	forest - edge	occasional	insectivore	arboreal	20	2010
White-whiskered Puffbird	*Malacoptila panamensis*	No	forest - interior	common	insectivore	arboreal	5	-
Gray-cheeked Nunlet	*Nonnula frontalis*	Yes	forest - interior	rare	insectivore	arboreal	30	1950
White-fronted Nunbird	*Monasa morphoeus*	Yes	forest - interior	rare	insectivore	arboreal	25	1950
Spot-crowned Barbet	*Capito maculicoronatus*	Yes	forest - edge	rare	insectivore	arboreal	32	1920
Collared Aracari	*Pteroglossus torquatus*	No	forest - edge	occasional	omnivore	arboreal	0	-
Keel-billed Toucan	*Ramphastos sulfuratus*	No	forest - edge	common	omnivore	arboreal	0	-
Yellow-throated Toucan	*Ramphastos ambiguus*	No	forest - edge	common	omnivore	arboreal	15	-
Black-cheeked Woodpecker	*Melanerpes pucherani*	No	forest - edge	common	insectivore	understory	10	-
Red-crowned Woodpecker	*Melanerpes rubricapillus*	Yes	edge	rare	insectivore	understory	0	1960
Cinnamon Woodpecker	*Celeus loricatus*	Yes	forest - interior	occasional	insectivore	understory	15	2000
Lineated Woodpecker	*Dryocopus lineatus*	No	forest - edge	occasional	insectivore	understory	0	-
Crimson-crested Woodpecker	*Campephilus melanoleucos*	No	forest - interior	occasional	insectivore	understory	0	-
Barred Forest-Falcon	*Micrastur ruficollis*	No	forest - interior	occasional	raptor	raptorial	10	Predicted
Collared Forest-Falcon	*Micrastur semitorquatus*	No	forest - interior	occasional	raptor	raptorial	3	-

Red-throated Caracara	*Ibycter americanus*	Yes	forest - edge	rare	raptor	raptorial	18	1990
Yellow-headed Caracara	*Milvago chimachima*	No	open	rare	raptor	raptorial	0	-
Bat Falcon	*Falco rufigularis*	No	forest - edge	rare	raptor	raptorial	0	-
Orange-chinned Parakeet	*Brotogeris jugularis*	No	edge	common	omnivore	arboreal	0	-
Brown-hooded Parrot	*Pyrilia haematotis*	No	forest - edge	rare	granivore	arboreal	15	-
Blue-headed Parrot	*Pionus menstruus*	No	forest - edge	common	granivore	arboreal	0	-
Red-lored Parrot	*Amazona autumnalis*	No	forest - edge	common	granivore	arboreal	0	-
Mealy Parrot	*Amazona farinosa*	No	forest - edge	common	granivore	arboreal	10	-
Yellow-crowned Parrot	*Amazona ochrocephala*	No	edge	rare	granivore	arboreal	0	-
Fasciated Antshrike	*Cymbilaimus lineatus*	Yes	forest - edge	rare	insectivore	arboreal	20	1970
Barred Antshrike	*Thamnophilus doliatus*	Yes	edge	rare	insectivore	arboreal	0	1970
Black-crowned Antshrike	*Thamnophilus atrinucha*	No	forest - interior	common	insectivore	arboreal	2	-
Spot-crowned Antvireo	*Dysithamnus puncticeps*	No	forest - interior	occasional	insectivore	arboreal	12	Predicted
Moustached Antwren	*Myrmotherula ignota*	Yes	forest - interior	rare	insectivore	arboreal	18	1930
Pacific Antwren	*Myrmotherula pacifica*	Yes	forest - edge	rare	insectivore	arboreal	20	1950
White-flanked Antwren	*Myrmotherula axillaris*	No	forest - interior	common	insectivore	arboreal	2	-
Checker-throated Stipplethroat	*Epinecrophylla fulviventris*	No	forest - interior	common	insectivore	arboreal	5	-
Dot-winged Antwren	*Microrhopias quixensis*	No	forest - interior	common	insectivore	arboreal	3	-
Dusky Antbird	*Cercomacroides tyrannina*	No	forest - edge	occasional	insectivore	arboreal	0	-

White-bellied Antbird	*Myrmeciza longipes*	Yes	forest - edge	rare	insectivore	terrestrial	0	1970
Chestnut-backed Antbird	*Myrmeciza exsul*	No	forest - interior	common	insectivore	terrestrial	9	-
Spotted Antbird	*Hylophylax naevioides*	No	forest - interior	common	insectivore	terrestrial	2	-
Bicolored Antbird	*Gymnopithys bicolor*	No	forest - interior	common	insectivore	terrestrial	5	-
Ocellated Antbird	*Phaenostictus mcleannani*	Yes	forest - interior	occasional	insectivore	terrestrial	18	1970
Streak-chested Antpitta	*Hylopezus perspicillatus*	Yes	forest - interior	rare	insectivore	terrestrial	18	1970
Black-faced Antthrush	*Formicarius analis*	Yes	forest - interior	occasional	insectivore	terrestrial	3	1950
Scaly-throated Leaftosser	*Sclerurus guatemalensis*	No	forest - interior	occasional	insectivore	terrestrial	2	-
Plain-brown Woodcreeper	*Dendrocincla fuliginosa*	No	forest - interior	common	insectivore	understory	5	-
Wedge-billed Woodcreeper	*Glyphorynchus spirurus*	No	forest - interior	occasional	insectivore	understory	10	-
Northern Barred-Woodcreeper	*Dendrocolaptes sanctithomae*	No	forest - interior	occasional	insectivore	understory	5	2010
Cocoa Woodcreeper	*Xiphorhynchus susurrans*	No	forest - interior	common	insectivore	understory	0	-
Black-striped Woodcreeper	*Xiphorhynchus lachrymosus*	No	forest - interior	common	insectivore	understory	18	-
Red-billed Scythebill	*Campylorhamphus trochilirostris*	Yes	forest - interior	rare	insectivore	understory	35	1930
Plain Xenops	*Xenops minutus*	No	forest - interior	common	insectivore	understory	0	-
Buff-throated Foliage-gleaner	*Automolus ochrolaemus*	Yes	forest - interior	rare	insectivore	arboreal	19	1960

Brown-capped Tyrannulet	*Ornithion brunneicapillus*	No	forest - edge	common	insectivore	arboreal	0	-
Southern Beardless-Tyrannulet	*Camptostoma obsoletum*	No	forest - edge	common	insectivore	arboreal	0	-
Yellow Tyrannulet	*Capsiempis flaveola*	No	edge	rare	insectivore	arboreal	0	-
Yellow-crowned Tyrannulet	*Tyrannulus elatus*	No	forest - edge	common	omnivore	arboreal	0	-
Forest Elaenia	*Myiopagis gaimardii*	No	forest - edge	common	insectivore	arboreal	3	-
Yellow-bellied Elaenia	*Elaenia flavogaster*	Yes	edge	occasional	insectivore	arboreal	0	1970
Lesser Elaenia	*Elaenia chiriquensis*	No	edge	occasional	insectivore	arboreal	0	-
Ochre-bellied Flycatcher	*Mionectes oleagineus*	No	forest - interior	common	omnivore	arboreal	0	-
Mistletoe Tyrannulet	*Zimmerius parvus*	No	forest - edge	common	omnivore	arboreal	0	-
Northern Scrub-Flycatcher	*Sublegatus arenarum*	No	edge	rare	insectivore	arboreal	0	-
Black-capped Pygmy-Tyrant	*Myiornis atricapillus*	No	forest - interior	common	insectivore	arboreal	15	-
Southern Bentbill	*Oncostoma olivaceum*	No	forest - edge	common	insectivore	arboreal	0	-
Slate-headed Tody-Flycatcher	*Poecilotriccus sylvia*	No	edge	rare	insectivore	arboreal	0	-
Common Tody-Flycatcher	*Todirostrum cinereum*	No	edge	common	insectivore	arboreal	0	-
Brownish Twistwing	*Cnipodectes subbrunneus*	Yes	forest - interior	rare	insectivore	arboreal	18	1950
Olivaceous Flatbill	*Rhynchocyclus olivaceus*	No	forest - interior	occasional	insectivore	arboreal	5	-

Yellow-olive Flycatcher	*Tolmomyias sulphurescens*	No	forest - edge	rare	insectivore	arboreal	0	-
Yellow-margined Flycatcher	*Tolmomyias assimilis*	No	forest - edge	common	insectivore	arboreal	2	-
Golden-crowned Spadebill	*Platyrinchus coronatus*	No	forest - interior	occasional	insectivore	arboreal	3	Predicted
Royal Flycatcher	*Onychorhynchus coronatus*	Yes	forest - interior	rare	insectivore	arboreal	10	1930
Ruddy-tailed Flycatcher	*Terenotriccus erythrurus*	No	forest - interior	occasional	insectivore	arboreal	0	-
Sulphur-rumped Flycatcher	*Myiobius sulphureipygius*	Yes	forest - interior	rare	insectivore	arboreal	25	1930
Black-tailed Flycatcher	*Myiobius atricaudus*	Yes	forest - edge	rare	insectivore	arboreal	5	1930
Tropical Pewee	*Contopus cinereus*	No	edge	rare	insectivore	arboreal	0	-
Long-tailed Tyrant	*Colonia colonus*	Yes	forest - edge	occasional	insectivore	arboreal	29	1990
Bright-rumped Attila	*Attila spadiceus*	No	forest - interior	common	insectivore	arboreal	3	-
Rufous Mourner	*Rhytipterna holerythra*	No	forest - interior	common	omnivore	arboreal	10	-
Dusky-capped Flycatcher	*Myiarchus tuberculifer*	No	forest - edge	common	insectivore	arboreal	0	-
Panama Flycatcher	*Myiarchus panamensis*	No	forest - edge	rare	insectivore	arboreal	0	-
Great Kiskadee	*Pitangus sulphuratus*	No	edge	rare	insectivore	arboreal	0	-
Boat-billed Flycatcher	*Megarynchus pitangua*	No	edge	occasional	insectivore	arboreal	0	-
Rusty-margined Flycatcher	*Myiozetetes cayanensis*	No	edge	common	insectivore	arboreal	0	-
Social Flycatcher	*Myiozetetes similis*	No	edge	common	insectivore	arboreal	0	-

Gray-capped Flycatcher	*Myiozetetes granadensis*	No	forest - edge	rare	insectivore	arboreal	25	-
White-ringed Flycatcher	*Conopias albovittatus*	No	forest - edge	rare	insectivore	arboreal	29	-
Streaked Flycatcher	*Myiodynastes maculatus*	No	edge	occasional	insectivore	arboreal	0	-
Piratic Flycatcher	*Legatus leucophaius*	No	edge	occasional	omnivore	arboreal	0	-
Tropical Kingbird	*Tyrannus melancholicus*	No	edge	common	insectivore	arboreal	0	-
Russet-winged Schiffornis	*Schiffornis stenorhyncha*	No	forest - interior	rare	omnivore	arboreal	5	Predicted
Speckled Mourner	*Laniocera rufescens*	No	forest - interior	rare	omnivore	arboreal	18	Predicted
Masked Tityra	*Tityra semifasciata*	No	forest - edge	occasional	omnivore	arboreal	0	-
Black-crowned Tityra	*Tityra inquisitor*	Yes*	forest - edge	rare	omnivore	arboreal	25	-
Cinnamon Becard	*Pachyramphus cinnamomeus*	No	edge	rare	insectivore	arboreal	5	-
White-winged Becard	*Pachyramphus polychopterus*	No	edge	occasional	insectivore	arboreal	5	-
Purple-throated Fruitcrow	*Querula purpurata*	No	forest - edge	common	omnivore	arboreal	3	-
Blue Cotinga	*Cotinga nattererii*	No	forest - edge	rare	frugivore	arboreal	15	-
Rufous Piha	*Lipaugus unirufus*	Yes	forest - interior	occasional	omnivore	arboreal	20	1990
Lance-tailed Manakin	*Chiroxiphia lanceolata*	Yes	forest - edge	rare	omnivore	arboreal	0	1990
White-ruffed Manakin	*Corapipo altera*	No	forest - interior	rare	omnivore	arboreal	30	-
Golden-collared Manakin	*Manacus vitellinus*	No	forest - edge	occasional	frugivore	arboreal	0	-

Red-capped Manakin	*Ceratopipra mentalis*	No	forest - interior	common	frugivore	arboreal	2	-
Scrub Greenlet	*Hylophilus flavipes*	Yes	forest - edge	rare	insectivore	arboreal	0	2000
Green Shrike-Vireo	*Vireolanius pulchellus*	Yes	forest - edge	rare	insectivore	arboreal	2	1990
Lesser Greenlet	*Pachysylvia decurtata*	No	forest - edge	common	insectivore	arboreal	2	-
Golden-fronted Greenlet	*Pachysylvia aurantiifrons*	No	forest - edge	rare	insectivore	arboreal	2	-
Yellow-green Vireo	*Vireo flavoviridis*	No	edge	rare	insectivore	arboreal	0	-
Black-chested Jay	*Cyanocorax affinis*	Yes	forest - edge	rare	omnivore	arboreal	3	1970
Scaly-breasted Wren	*Microcerculus marginatus*	Yes	forest - interior	rare	insectivore	terrestrial	15	1930
House Wren	*Troglodytes aedon*	No	edge	rare	insectivore	arboreal	0	-
White-headed Wren	*Campylorhynchus albobrunneus*	Yes	forest - interior	rare	insectivore	arboreal	35	1950
Black-bellied Wren	*Pheugopedius fasciatoventris*	Yes	forest - interior	rare	insectivore	arboreal	15	1960
Isthmian Wren	*Cantorchilus elutus*	No	open	rare	insectivore	arboreal	0	-
Bay Wren	*Cantorchilus nigricapillus*	Yes	forest - edge	rare	insectivore	arboreal	25	1950
Buff-breasted Wren	*Cantorchilus leucotis*	Yes	forest - edge	rare	insectivore	arboreal	5	1950
White-breasted Wood-Wren	*Henicorhina leucosticta*	Yes	forest - interior	occasional	insectivore	terrestrial	3	1950
Song Wren	*Cyphorhinus phaeocephalus*	Yes	forest - interior	occasional	insectivore	terrestrial	15	1960
Long-billed Gnatwren	*Ramphocaenus melanurus*	Yes	forest - edge	occasional	insectivore	arboreal	2	1990
Tropical Gnatcatcher	*Polioptila plumbea*	No	forest - edge	common	insectivore	arboreal	0	-

Clay-colored Thrush	*Turdus grayi*	No	edge	rare	omnivore	arboreal	0	-
White-throated Thrush	*Turdus assimilis*	No	forest - edge	rare	omnivore	arboreal	25	-
Tropical Mockingbird	*Mimus gilvus*	No	edge	rare	omnivore	arboreal	2	-
Yellow-crowned Euphonia	*Euphonia luteicapilla*	No	edge	rare	frugivore	arboreal	5	-
Thick-billed Euphonia	*Euphonia laniirostris*	No	edge	occasional	frugivore	arboreal	5	-
Fulvous-vented Euphonia	*Euphonia fulvicrissa*	No	forest - edge	occasional	omnivore	arboreal	0	-
White-vented Euphonia	*Euphonia minuta*	Yes	forest - edge	occasional	frugivore	arboreal	29	2010
Rosy Thrush-Tanager	*Rhodinocichla rosea*	Yes	forest - edge	rare	insectivore	terrestrial	5	1930
Orange-billed Sparrow	*Arremon aurantiirostris*	No	forest - edge	rare	omnivore	terrestrial	3	-
Black-striped Sparrow	*Arremonops conirostris*	Yes	edge	rare	omnivore	terrestrial	0	1990
Yellow-billed Cacique	*Amblycercus holosericeus*	No	open	rare	omnivore	arboreal	3	-
Crested Oropendola	*Psarocolius decumanus*	No	forest - edge	rare	omnivore	arboreal	20	-
Chestnut-headed Oropendola	*Psarocolius wagleri*	No	forest - edge	common	omnivore	arboreal	3	-
Scarlet-rumped Cacique	*Cacicus uropygialis*	No	forest - interior	rare	omnivore	arboreal	15	-
Yellow-rumped Cacique	*Cacicus cela*	No	forest - edge	common	omnivore	arboreal	3	-
Yellow-backed Oriole	*Icterus chrysater*	No	forest - edge	occasional	omnivore	arboreal	3	-
Yellow-tailed Oriole	*Icterus mesomelas*	Yes	forest - edge	rare	omnivore	arboreal	15	1940
Giant Cowbird	*Molothrus oryzivorus*	No	forest - edge	occasional	omnivore	terrestrial	5	-

Great-tailed Grackle	*Quiscalus mexicanus*	No	open	rare	omnivore	terrestrial	0	-
Buff-rumped Warbler	*Myiothlypis fulvicauda*	Yes	forest - interior	rare	insectivore	terrestrial	20	1960
Rufous-capped Warbler	*Basileuterus rufifrons*	Yes	forest - edge	occasional	insectivore	arboreal	3	1960
Dusky-faced Tanager	*Mitrospingus cassinii*	Yes	forest - edge	rare	insectivore	arboreal	25	1970
Red-throated Ant-Tanager	*Habia fuscicauda*	No	forest - edge	rare	omnivore	arboreal	3	-
Blue-black Grosbeak	*Cyanocompsa cyanoides*	No	forest - edge	occasional	omnivore	arboreal	3	-
Blue-gray Tanager	*Thraupis episcopus*	No	edge	common	omnivore	arboreal	0	-
Palm Tanager	*Thraupis palmarum*	No	edge	common	omnivore	arboreal	0	-
Golden-hooded Tanager	*Tangara larvata*	No	forest - edge	common	omnivore	arboreal	0	-
Plain-colored Tanager	*Tangara inornata*	No	edge	common	omnivore	arboreal	0	-
Bay-headed Tanager	*Tangara gyrola*	No	forest - edge	rare	omnivore	arboreal	20	-
Green Honeycreeper	*Chlorophanes spiza*	No	forest - edge	common	omnivore	arboreal	0	-
Sulphur-rumped Tanager	*Heterospingus rubrifrons*	No	forest - edge	occasional	omnivore	arboreal	30	Predicted
Blue-black Grassquit	*Volatinia jacarina*	No	open	occasional	omnivore	arboreal	0	-
Gray-headed Tanager	*Eucometis penicillata*	No	forest - interior	occasional	omnivore	arboreal	0	-
White-shouldered Tanager	*Tachyphonus luctuosus*	No	forest - edge	common	omnivore	arboreal	2	-
White-lined Tanager	*Tachyphonus rufus*	Yes	open	rare	omnivore	arboreal	3	1960
Flame-rumped Tanager	*Ramphocelus flammigerus*	Yes	edge	rare	omnivore	arboreal	20	1960

Crimson-backed Tanager	*Ramphocelus dimidiatus*	No	edge	occasional	omnivore	arboreal	0	-
Shining Honeycreeper	*Cyanerpes lucidus*	No	forest - edge	common	frugivore	arboreal	15	-
Red-legged Honeycreeper	*Cyanerpes cyaneus*	No	forest - edge	common	frugivore	arboreal	0	-
Scarlet-thighed Dacnis	*Dacnis venusta*	No	forest - edge	occasional	omnivore	arboreal	29	Predicted
Blue Dacnis	*Dacnis cayana*	No	forest - edge	common	omnivore	arboreal	0	-
Bananaquit	*Coereba flaveola*	No	edge	rare	omnivore	arboreal	10	-
Yellow-faced Grassquit	*Tiaris olivaceus*	No	edge	rare	omnivore	arboreal	30	-
Thick-billed Seed-Finch	*Sporophila funerea*	No	edge	rare	granivore	arboreal	0	-
Variable Seedeater	*Sporophila corvina*	No	edge	common	granivore	arboreal	0	-
Slate-colored Seedeater	*Sporophila schistacea*	No	forest - edge	rare	granivore	arboreal	0	-
Yellow-bellied Seedeater	*Sporophila nigricollis*	No	open	occasional	granivore	arboreal	0	-
Black-headed Saltator	*Saltator atriceps*	No	edge	rare	omnivore	arboreal	29	-
Buff-throated Saltator	*Saltator maximus*	Yes	edge	rare	omnivore	arboreal	3	1960
Slate-colored Grosbeak	*Saltator grossus*	No	forest - interior	occasional	omnivore	arboreal	18	Predicted
Streaked Saltator	*Saltator striatipectus*	Yes	edge	rare	omnivore	arboreal	3	1970

***** The status of missing species marked “Yes” with an asterisk is uncertain. Populations of these species appear functionally extirpated, but poor detection rates may allow them to persist in small, unencountered populations on the island. These birds were omitted from data summaries and logistic regressions.

† “Predicted” is used in the Last Decade column to indicate remaining species with low abundances and declining populations that share traits with extirpated species and are considered most likely to go extinct on BCI in the coming decades.

Table S4. Residency status of non-aquatic, non-aerial species first detected on BCI after 1950 sorted by decade of first detection. Residency categories include “vagrant/flyover”: species unlikely to maintain stable, resident breeding populations on BCI; “ephemeral”: transitory breeders that maintain only intermittent breeding populations; “expanding”: birds experiencing range expansions along the canal but not yet permanent residents of BCI - often urban associated; and “colonized”: new species with stable, permanent breeding populations. See Table S3 for corresponding scientific names.

**Common Name**	**Status**	**Decade**
Violaceous Quail-Dove	Ephemeral	1950
Gray-lined Hawk	Vagrant	1950
Great Kiskadee	Colonized	1950
White-throated Thrush	Ephemeral	1950
Tropical Mockingbird	Expanding	1950
Great-tailed Grackle	Expanding	1950
Slate-colored Seedeater	Ephemeral	1950
Brown-hooded Parrot	Vagrant	1990
White-tailed Kite	Vagrant	1990
White-ruffed Manakin	Ephemeral	1990
Golden-fronted Greenlet	Ephemeral	1990
Yellow-crowned Euphonia	Ephemeral	1990
Orange-billed Sparrow	Vagrant	1990
Scarlet-rumped Cacique	Vagrant	1990
Yellow-faced Grassquit	Ephemeral	1990
Pearl Kite	Vagrant	2000
Bicolored Hawk	Vagrant	2000
Yellow-headed Caracara	Expanding	2000

Table S5. Average southern range limit between missing and extant bird species on BCI by habitat association (with SD in parentheses), with the calculated difference between average missing and extant values and the percent of the 65 km-long central Panama isthmus that the difference in averages represents.

Habitat Association	Average Southern Limit (km)		Difference (km)	Percent of Isthmus
	Missing	Extant		
All habitats	13.8 (± 11.2)	5.7 (± 8.5)	8.1	12.5%
All forest-associated	16.8 (± 10.3)	7.0 (± 8.7)	9.8	15.1%
Forest interior	19.3 (± 8.2)	7.4 (± 7.9)	11.9	18.3%
Forest edge	14.6 (± 11.6)	6.7 (± 9.4)	7.9	12.2%
Edge	3.5 (± 7.4)	2.9 (± 7.8)	0.6	0.9%
Open	3.0 (± 0.0)	0.33 (± 1.0)	2.7	4.1%

Table S6. Mean environmental factor values for each mainland subregion cluster identified using hierarchical cluster analysis (with ranges italicized in parentheses). See Table S2 for definitions of environmental factors.

Cluster	# Subregions	Bird Richness	Forest Age	Altitude (m)	Area (km^2)	% Forest	% Unfragmented	% Urban	Plant Richness	Precip (mm/yr)
1	5	150.2 (147-156)	2.4	231.6 (109-349)	47.7 (11.2-78.5)	67.0 (43.0-91.0)	88 (80-99)	0.4 (0-1)	74.0 (67-85)	2338.2 (2100-2550)
2	8	237.9 (220-271)	2.5	204.6 (111-278)	49.6 (20.0-74.0)	78.9 (46.2-94.8)	95 (78-100)	0.7 (0.1-3.1)	81.9 (57-96)	2623.1 (1935-3250)
PRO	1	120	2	137	17.28	63	81	1.1	109	3000

Table S7. Abbreviated codes and full names of physiogeographic subregions in alphabetical order by code. Subregion names are based primarily on politically administered counties (*corregimientos*) in the Canal area.

**Code**	**Full Name**
AN	Achiote North
ANC	Ancon
ARJ	Araijan
AS	Achiote South
BCI	Barro Colorado Island
CAT	Cativa
CLA	Santa Clara
CRIS	Cristobal
EMP	Nuevo Emperador
FAR	Farfan
GAL	Galeta
GIG	Gigante
LASC	Las Cruces
MAN	Mandinga
NSO_M	North Soberania - Mid
NSO_N	North Soberania - North
NSO_S	North Soberania - South
PENIN	Barro Colorado National Monument Peninsula
PRO	Nueva Providencia
PTY	Panama City
ROD	Rodman
SIL	Silvestre
SLO	San Lorenzo
SSOB	South Soberania
VER	Veracruz
